# Temporal evolution of digital health communication in Rheumatoid Arthritis: A longitudinal NLP analysis of reddit discussions (2018–2024)

**DOI:** 10.1371/journal.pone.0341006

**Published:** 2026-01-20

**Authors:** Naisarg Patel, Rajesh Sharma, Prakash Lingasamy, Vino Sundararajan, Sajitha Lulu S, Vijayachitra Modhukur

**Affiliations:** 1 Integrative Multiomics Lab, School of Bio Sciences and Technology, Vellore Institute of Technology, Vellore, Tamil Nadu, India; 2 School of AI and Computer Science, Plaksha University, Punjab, India; 3 Institute of Computer Science, University of Tartu, Tartu, Estonia; 4 Department of Obstetrics and Gynecology, Institute of Clinical Medicine, University of Tartu, Tartu, Estonias; 5 Nalam Biosciences OÜ, Tartu, Estonia; Hormozgan University of Medical Sciences, IRAN, ISLAMIC REPUBLIC OF

## Abstract

Rheumatoid arthritis (RA) is a chronic autoimmune condition characterized by joint pain, fatigue, and reduced quality of life. Although pharmacological interventions, such as non-steroidal anti-inflammatory drugs (NSAIDs) and disease-modifying antirheumatic drugs (DMARDs), address physical symptoms, the psychological and emotional challenges associated with RA are frequently neglected. Social media platforms, particularly Reddit, have emerged as significant venue for patients to share experiences and seek support, a trend that has intensified during the COVID-19 pandemic. This study examined six years (2018–2024) of discussions from the r/rheumatoid and r/rheumatoidarthritis subreddits, encompassing 22,537 posts and 276,209 comments. Natural language processing (NLP) techniques were implemented to analyze sentiment, emotions, discussion topics, drug mentions, and hyperlink-sharing patterns across three phases: pre-COVID, during COVID, and post-COVID. The analysis indicated that comments were predominantly positive, whereas posts exhibited increased negativity following the onset of COVID-19. Fear and sadness were prevalent in posts, while comments frequently conveyed joy, underscoring the community’s supportive nature. Topic modeling identified recurring discussions concerning treatment efficacy, mental health, and pandemic-related disruptions. Methotrexate emerged as the most frequently mentioned medication, with notable emotional variation during the pandemic. Hyperlink patterns suggested an increasing reliance on medical and academic sources, reflecting patients’ need for reliable information. These findings illustrate how online communities capture evolving patient experiences and unmet needs. Insights from such discussions can inform healthcare providers, policymakers, and public health communicators in developing patient-centered strategies that address both the emotional and informational dimensions of RA care.

## 1. Introduction

Rheumatoid arthritis (RA) is one of the most prevalent chronic autoimmune diseases and is characterized by persistent joint inflammation, often leading to progressive joint damage and disability. RA affects approximately 5 per 1,000 adults globally, causing severe physical, psychological, and economic burdens on patients and society [1,2]. Notably, women are more prone to developing RA compared to men. Common symptoms such as joint pain, stiffness, fatigue, and loss of mobility are frequently accompanied by psychological issues such as anxiety, depression, and feelings of helplessness [3,4]. These challenges not only reduce patient quality of life and place a significant burden on families but also contribute to decreased workplace productivity and rising healthcare costs. Current RA treatments, including disease-modifying antirheumatic drugs (DMARDs) and biologics, aim to alleviate symptoms rather than the root causes of RA [5,6]. To better understand the lived experiences and treatment perceptions of RA with patients, it is essential to explore settings beyond traditional clinical environments [7,8].

Traditional survey-based studies of RA patient experiences offer valuable insights but often suffer from small sample sizes and limited scopes, potentially overlooking critical patient concerns such as mental health and emotional well-being [9,10]. In contrast, online platforms such as Reddit and X (formerly Twitter) offer access to large-scale, real-time, patient-driven data [11,12]. While X.com (formerly Twitter) facilitates concise communication, it limits the depth of emotional expressions [13]. In comparison, Reddit enables longer, context-rich discussions that capture nuanced emotional and psychological dimensions of RA treatment experiences [13–17]. Emerging natural language processing (NLP) methods, such as structural topic modeling (STM) and sentiment analysis, have proven effective in analyzing online discourse, from COVID-19 vaccine hesitancy to caregiver challenges in dementia [18–20]. Previous studies, such as those on RheumTwitter, highlight the role of social media in public health research but often overlook Reddit’s value in identifying key recurring themes, such as mental health, treatment efficacy, and emotional toll of living with RA [14,16,17,21–25]. Moreover, most prior research has focused on cross-sectional analyses, neglecting longitudinal trends in patient experiences that evolve over time, particularly in response to significant events like the COVID-19 pandemic[26,27]. Additionally, dynamics such as drug-related discussions, hyperlink-sharing behavior, and emerging themes related to treatment efficacy and mental health have not been comprehensively studied, particularly in the context of the pandemic’s impact on healthcare access and patient concerns [28–30].

Accordingly, this study is guided by three research questions:

R1: How do sentiments and emotional expressions related to RA change across pre-COVID, COVID, and post-COVID periods?

R2: What thematic shifts emerge in discussions about symptoms, medication experiences, mental health, and social support across these phases?

R3: How do drug-mention patterns and hyperlink-sharing behaviors evolve over time?

This study employs three complementary frameworks. Social Support Theory explains how individuals exchange emotional and informational support in online communities, showing patterns between help-seeking posts and supportive responses [31,32]. Digital Health Literacy frameworks uncover hyperlink-sharing behaviors by showing how users evaluate online health information, particularly during the COVID-19 pandemic when patients relied on online resources [33]. Theories of emotional expression demonstrate how anonymity enables candid disclosures while fostering community support, evident in the contrast between distressed posts and solution-oriented comments [34]. These frameworks integrate the study within digital health communication and online social behavior, guiding our analysis of sentiment, emotional expression, and information-seeking behavior.

Building on these foundations, our study applies NLP and STM techniques to Reddit discussions on RA across pre-COVID, COVID, and post-COVID periods (2018–2024). We analyze sentiment and emotion trends, including positive, neutral, and negative states, while examining feelings like anxiety and frustration. On the other hand, topic modeling identifies themes in treatment efficacy, mental health, and social support. Our analysis examines drug-related discussions and patient concerns, while evaluating hyperlink-sharing to understand information-seeking behavior. The results may have practical implications for clinicians, psychologists, health communicators and moderators developing patient-centered digital care strategies.

## 2. Methodology

### 2.1 Data collection

The data was obtained using Project Arctic Shift [35], an open-source initiative that provides public access to Reddit data, posts, and comments. This tool enables options for filtering by specific subreddits and time intervals. This study collected data from subreddits r/rheumatoid and r/rheumatoidarthritis from September 2018 to October 2024. The descriptions of the subreddits mention “autoimmune arthritis sufferers” and “people living with RA,” indicating that the majority of users in both subreddits may have been suffering from RA. The resulting data was extracted and downloaded in JavaScript Object Notation Lines (JSONL) format and organized further based on relevant subreddits and periods, resulting in the dataset for analysis. The data was categorized into three distinct periods: Pre-COVID (from 11 September 2018–10 March 2020, spanning 1 year and 6 months), COVID (from 11 March 2020–4 May 2023, covering 3 years, 1 month, and 24 days), and Post-COVID (from 5 May 2023–4 November 2024, lasting 1 year and 6 months). This study utilized only publicly available data from Reddit, a pseudonymous platform where users can be recognized over time by their usernames, their actual identities remain undisclosed.

To prepare the dataset for further analysis, the JSONL files from the previous step were converted to CSV (Comma Separated Values) which facilitates more accessible analysis using Python and Microsoft Excel.

The following processing steps were adapted from Goel et al. [36] and performed:

i) **Data Cleaning:** We removed posts and comments deleted by moderators and authors. Additionally, comments whose parent posts were deleted were excluded from the dataset.
ii) **Text Cleaning and Normalization:** We converted all text to lowercase and further removed punctuations, symbols, hyperlinks, email addresses, and hashtags (starting with #). Further, words stretched for emphasis (e.g., “goooood” to “good”) were normalized to their general form. Additionally, abbreviations commonly used in social media were expanded to their full forms.
iii) **Stop Word Removal:** Non-essential words (e.g., “and,” “the”) that do not contribute to overall sentiment were removed.
iv) **Spelling Correction and Lemmatization:** The Spellchecker library [37] is utilized to identify and correct spelling errors in the text. We then apply lemmatization using the NLTK package. Lemmatization reduces words to their base form (lemma), focusing on linguistic correctness. For instance, the root of the word “running” is “run” in lemmatization. Specifically, we use the NLTK’s WordNetLemmatizer for lemmatization.

### 2.2 Sentiment and emotion analysis

In our study we considered two widely used methods for sentiment analysis: Lexicon Based Approach and Machine Learning Approach [38]. To determine the optimal approach, 1,000 randomly selected posts and comments were manually labeled for sentiment analysis. The labels were independently verified by the authors, (VM and PL). The latter was used to fine-tune the Robustly optimized BERT approach (RoBERTa), a pre-trained language model [39]. The performance of RoBERTa was compared to the evaluation of Natural Language Toolkit’s (NLTK) Valence Aware Dictionary and sEntiment Reasoner (VADER) [40]. The models were evaluated using metrics such as Accuracy, Precision, Recall and F1-score implemented using Scikit-learn library [41].

Sentiments classified by RoBERTa were categorized as Negative (0), Neutral (1), and Positive (2), while VADER utilized compound scores ranging from −1 to +1 to classify sentiments as Negative (−1 to −0.05), Neutral (−0.05 to +0.05), or Positive (+0.05 to +1). For emotion analysis, Jochen Hartman’s pre-trained model [42], accessible via Hugging Face, was implemented to predict Ekman’s six basic emotions, namely, anger, disgust, fear, joy, sadness, and surprise along with a neutral category. Notably, Jochen Hartman’s model reported an accuracy of 66%, compared with a random-chance baseline of 14% [42], and it has been applied in several previous studies on emotion analysis studies [43,44]. Subsequently, each post and comment was assigned the emotion label with the highest probability score from the emotion analysis model. To evaluate whether the differences in user expression were statistically significant, we performed Chi-square tests of independence to analyze the distribution of sentiment categories (positive, neutral, negative) and emotion categories across the pre-, during-, and post-COVID periods [45].

### 2.3 Theme and topic modeling

To identify prevalent themes and topics within the datasets, BERTopic [46], a topic modeling framework that combines multiple advanced techniques, was used. Here, the text data was initially converted into numerical embeddings using Sentence Transformer [47] and then reduced in dimensionality through Uniform Manifold Approximation and Projection (UMAP) [48]. The resulting reduced embeddings were clustered with Hierarchical Density-Based Spatial Clustering of Applications with Noise (HDBSCAN) [49]. Finally, the text corpus was transformed into a token count matrix using Scikit-learn’s CountVectorizer function [41], which served as input for BERTopic to extract the top 10 topics.

Additionally, word clouds were generated using the Python library wordcloud [50], which provided visualization of the most frequently used words in the dataset.

### 2.4 Comparative analysis of subreddits

To understand the similarities and differences in the discussions of r/rheumatoid and r/rheumatoidarthritis, we compared the posts and comments for the subreddits. The datasets for posts and comments across the time-periods were combined for each of the subreddits. These two datasets were then used to perform sentiment analysis, emotion analysis, and topic modeling as described above.

### 2.5 Drug mention analysis

Mentions of drugs in Reddit posts and comments were identified using a keyword matching approach. A list of drug names was created by cross-referencing approved rheumatoid arthritis (RA) medications from DrugBank [51]. A dictionary was made using the DrugBank database to include synonyms, generic and brand names for these drugs (e.g., “Methotrexate,” “Enbrel,” “Zantac”), linking the generic name to the other names to ensure comprehensive detection.

Temporal trends in drug mentions were analyzed across three distinct periods: pre-COVID, during COVID, and post-COVID. This segmentation allowed us to assess how external events, such as the pandemic, influenced discussions about RA treatments. For example, changes in the frequency of mentions of specific drugs or classes could highlight shifts in patient preferences, awareness, or availability of treatments during these periods.

The co-occurrence of drug mentions with other topics, such as sentimental and emotional expressions, was also examined. For instance, posts discussing drug efficacy may be found alongside terms reflecting fear or sadness. This analysis further enhances the understanding of how RA medications are perceived and discussed in the online community.

### 2.6 Hyperlink-based analysis

Hyperlinks embedded in posts and comments were systematically extracted and analyzed to identify the types of external resources shared within RA-related discussions. The hyperlinks were evaluated using the Python “requests” library. Those links that returned a 200 (OK) response were considered as a successful request and subject to further analysis.

Hyperlinks were categorized using GPT-4 (OpenAI, accessed 27 November 2024) [52]. Using the prompt “Classify the following list of website URLs into meaningful categories based on their primary purpose or content type into Academic Resources, Blogs and Personal Websites, Government Websites, Medical and Healthcare Websites, News Outlets, Non-Profit Organizations, Shopping, and Social Media platforms. Provide the output as a Python dictionary where each key is the category name and the value is a list of URLs belonging to that category”. A 20% stratified random sample of all URLs (N = 363) was independently reviewed by two co-authors to ensure the reliability of these AI classifications. Inter-rater agreement was quantified using Cohen’s kappa (κ), capturing the level of agreement between reviewers. This classification provided insights into the diversity and credibility of resources accessed by the RA community.

The temporal distribution of shared hyperlinks was analyzed across pre-COVID, during COVID, and post-COVID periods to detect changes in information-seeking behavior and resource preference. For example, an increased reliance on medical websites during the COVID period might reflect heightened concerns about treatment options or infection risks. Additionally, link-sharing trends were analyzed to evaluate the popularity and reliability of specific resources, such as frequently cited domains or pages. This analysis provided valuable insights into the types of external information the RA community relied upon and how these resources shaped discussions within the online space.

### 2.7 Ethical considerations

In accordance with the Research Ethics Committee policy of our institutes, this study did not require ethical approval. The analysis was conducted using publicly accessible pseudonymous data from Reddit. Consent from individuals or Reddit was not obtained, as the posts were publicly available, voluntarily shared, and did not contain identifiable personal information. Additionally, no individual user-based analysis was performed.

## 3. Results

### 3.1 Overview of the dataset

The initial dataset for our study, extracted from the Reddit subreddits r/rheumatoid and r/rheumatoidarthritis, comprised 26,800 posts and 302,969 comments (S1 Table in S1 File). Following processing steps, the final dataset contained 22,537 posts and 276,209 comments. Table 1 provides a detailed distribution of the dataset after processing.

**Table 1:** Dataset statistics after pre-processing.

Subreddit	r/rheumatoid		r/rheumatoidarthritis		Total	
Time	Posts	Comments	Posts	Comments	Posts	Comments
Pre-COVID	2583	31764	357	2544	2940	34308
COVID	6528	90540	3479	37038	10007	90577
Post-COVID	5764	71685	3826	42638	9590	114323
**Total**	**14875**	**193989**	**7662**	**82220**	**298746**	

**Fig 1:**
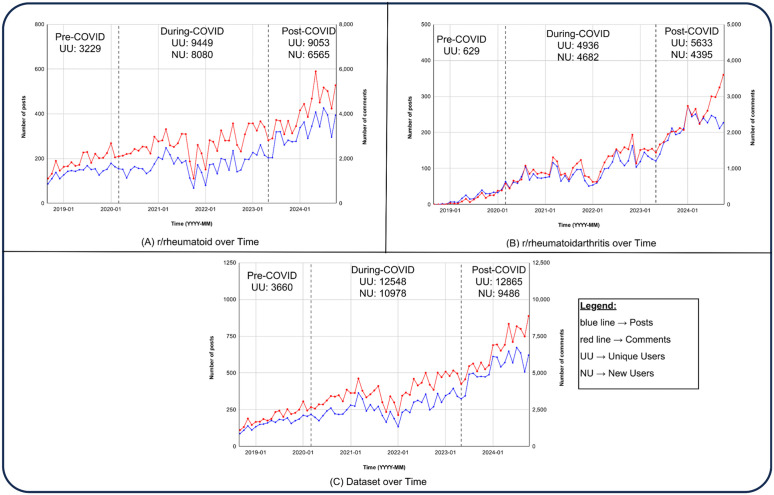
Number of Posts and Comments in the dataset across the time periods. (A) Post and Comments in the r/rheumatoid Subreddit (B) Post and Comments in the r/rheumatoidarthritis Subreddit (C) Combined Posts and Comments from both Subreddits. The blue line represents the posts, while the red line represents the number of comments.

To examine temporal trends, the frequency of posts and comments was plotted over time across the relevant subreddits (Fig 1). These visualizations revealed patterns of activity within the RA community, including spikes in discussion volume that corresponded to major events, such as the onset of the COVID-19 pandemic.

Our analysis showed that the subreddit r/rheumatoid had a higher number of users and user discussions (Fig 1A) compared to r/rheumatoidarthritis (Fig 1B). This may be because the subreddit r/rheumatoid was created in 2012, while r/rheumatoidarthritis was created only in 2018. However, the number of users in both subreddits has been increasing constantly (Fig 1C). Interestingly, subreddits gained a large number of users during the COVID period. The increase in access to the internet and platforms such as Reddit might also impact the increasing number of unique users. Notably, our analysis reveals a surge in new users during the COVID-19 pandemic, coinciding with global lockdown measures

### 3.2 Sentiment and emotion analysis

#### 3.2.1 Comparison of NLP and machine learning approach for sentiment analysis.

A comprehensive evaluation of the sentiment analysis approaches on our Reddit-based dataset revealed notable differences in performance. The accuracy, precision, recall and F1-Score for NLTK’s VADER was 89.70%, 89.72%, 89.70% and 0.90 respectively when compared to 82.50%, 82.65%, 82.50% and 0.82 for the Fine-Tuned RoBERTa model. Since NLTK’s VADER demonstrated superior performance, achieving a 7.2% higher accuracy compared to other methods, it was chosen for our study.

#### 3.2.2 Sentiment analysis.

**Fig 2:**
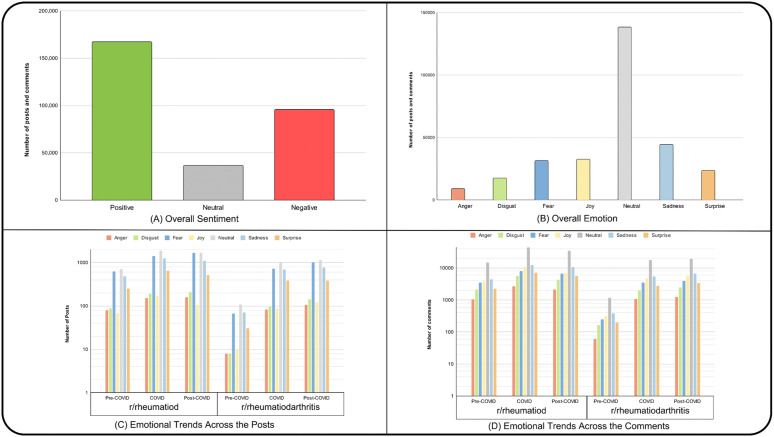
Overall sentiment and emotion across the dataset. (A) Sentiment classification showing the proportions of positive, neutral and negative (B) Emotion classification showing the proportions of anger, disgust, fear, joy, neutral, sadness, and surprise. (C) Emotional trends in posts: Distribution of emotions (anger, disgust, fear, joy, neutral, sadness, and surprise) in posts from r/rheumatoid and r/rheumatoidarthritis during the pre-COVID, COVID, and post-COVID periods. (D) Emotional trends in comments: Distribution of the same emotions in comments from the two subreddits during the same time periods. The y-axis represents the frequency of posts or comments expressing each emotion.

Sentiment analysis revealed that the majority of the dataset displayed positive sentiment (55.89%), a small number of sentiments were neutral (12.21%). At the same time, a significant number of sentiments were negative (31.90%) (Fig 2A). Table 2 shows the number, percentage, and examples of posts and comments categorized by sentiment type. Emotion analysis revealed that most of the dataset was associated with joy (Fig 2B). S2 Table in S1 File shows the number, percentage, and examples of posts and comments categorized by emotion type. In addition, fear and sadness were also prevalent, followed by surprise and disgust. Neutral emotion is often associated with questions or statements without any specific emotional tone.

**Table 2:** Examples of posts annotated as positive, negative, or neutral sentiments.

Sentiment	Total posts	Examples
Positive	166965 (55.89%)	• Oh wow, that’s amazing! Thank you for tip, I will definitely check out the company’s website:) Congrats on the success, that’s so wonderful:) • This! Some lovely cashmere gloves would help with the hand pain but also just be a lovely gift. Or honestly anything cashmere, lol. A paraffin wax bath could be a nice self-care gift that helps with the RA. • Sounds like great fun, I hope you had a great night. like you say, it’s about the small wins.
Neutral	36481 (12.21%)	• Do you prefer heat or cold? What else do you use? • What work accommodations have you guys asked for? • I am on 15 mg of methotrexate a week.
Negative	95300 (31.90%)	• I am wondering this exact same thing right now. It sucks. I hate hate hate it!!! I’m sad. I hurt. My kids mad. Mommy sucks a lot more than she used to. • I’m really worried I won’t find anyone because of all my health issues:(I’ve struggled so hard with relationships • Ah yes I tried meloxicam but it gave me horrid stomach cramps.

**Fig 3:**
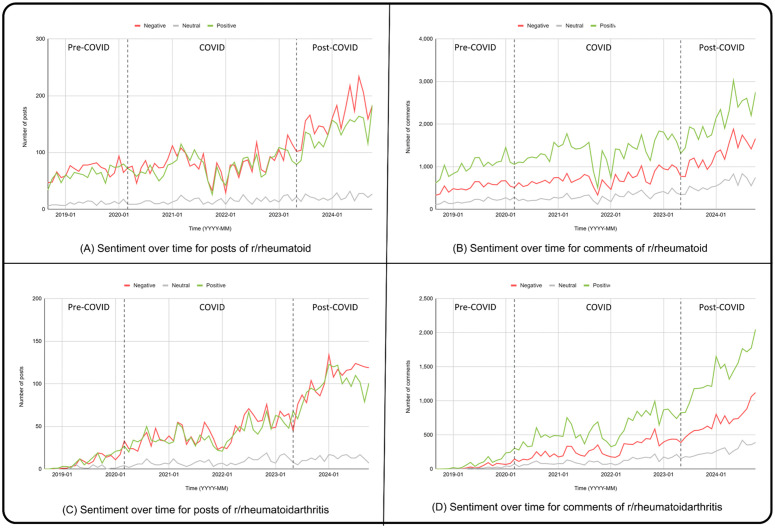
Sentiment analysis of Reddit posts and comments in r/rheumatoid and r/rheumatoidarthritis subreddits from September 2018 to October 2024. (A) Sentiment trends over time for posts in r/rheumatoid. (B) Sentiment trends over time for comments in r/rheumatoid. (C) Sentiment trends over time for posts in r/rheumatoidarthritis. (D) Sentiment trends over time for comments in r/rheumatoidarthritis. Vertical dashed lines mark the pre-COVID, COVID, and post-COVID periods. Sentiment is categorized as positive (green), neutral (gray), and negative (red).

Upon performing the sentiment analysis, we observed clear differences in the sentiments expressed between posts and comments. Specifically, posts exhibited a roughly balanced distribution of positive and negative sentiments, with a noticeable rise in negative sentiment during the post-COVID period (Fig 3A–3B). In contrast, comments remained predominantly positive across all time periods (Fig 3C–3D). A chi-square test of independence confirmed that sentiment distributions varied significantly across the pre-, during-, and post-COVID periods [45]. (χ²(4) = 353.57, p < 10 ⁻ ⁷³), indicating meaningful temporal shifts in user sentiment.

#### 3.3.3 Emotion analysis.

**Fig 4:**
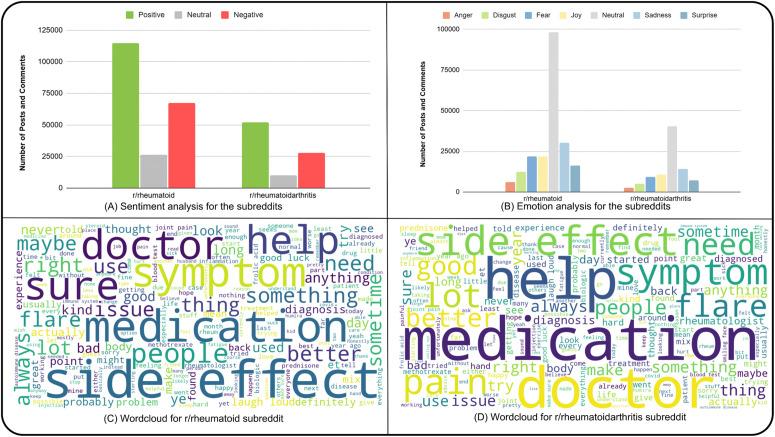
Analysis of Reddit posts and comments across the subreddits. (A) Sentiment analysis showing the distribution of positive, neutral, and negative sentiments in r/rheumatoid and r/rheumatoidarthritis. (B) Emotion analysis displaying the distribution of emotions (anger, disgust, fear, joy, neutral, sadness, and surprise) in both subreddits. (C) Word cloud for r/rheumatoid illustrating the most frequently used words. (D) Word cloud for r/rheumatoidarthritis highlighting prominent words based on frequency.

The emotional analysis of posts revealed that the posts are dominated by fear and sadness, followed by surprise. In contrast, only a few posts are classified as joyful (Fig 4A). On the other hand, the comments were dominated by joy, followed by fear and sadness (Fig 4B). This contrast may stem from newly diagnosed RA patients using Reddit to express their concerns and seek guidance, resulting in posts characterized by negative emotions such as fear or sadness. However, as a response, commenters often provided supportive messages and shared personal experiences, contributing to a more joyful emotional tone in the comments. Questions or statements without any specific emotional tone are classified as neutral and it’s common in the posts and comments. Anger and disgust are less prevalent due to the presence of moderators of the subreddits, they remove any hurtful or inappropriate posts. The distribution of sentiment varied significantly across the three periods which is shown by the χ²(4) = 400.91, p = 2.42 × 10^78^. The p-value is less than 0.001, thus observed emotion counts differed substantially from the expected frequencies under the null hypothesis of no association. This confirms that user emotion changed significantly across pandemic phases.

### 3.3 Theme and topic modeling

The word clouds show that discussions in both subreddits consistently revolve around “pain,” “doctor,” and “medication,” reflecting core concerns of individuals managing rheumatoid conditions (S1 Fig). During the COVID period, terms like “side effects,” “help,” and “symptom” became more prominent, suggesting heightened worries about treatment changes and symptom management. Post-COVID, the persistence of these terms indicates ongoing challenges and continued reliance on medical guidance. Tables 3 and 4 present the main topics extracted over various periods using BERTopic.

**Table 3:** Top 10 topics extracted from the posts and comments of r/rheumatoid subreddit across the time-periods.

r/rheumatoid		
Pre-COVID	COVID	Post-COVID
**Posts**		
Arthritis Diagnosis and Treatment	Rheumatoid Arthritis Diagnosis	Rheumatoid Arthritis Treatment
Rheumatoid Arthritis Symptoms	Joint Pain and Inflammation	Joint Pain and Inflammation
Medications for RA	Rheumatologist Treatments	Methotrexate and Biologics
	COVID-19 Vaccination	Chronic Illness Symptoms
		Prednisone for Arthritis
**Comments**		
Arthritis Diagnosis and Treatment	Arthritis Diagnosis and Treatment	Arthritis Symptoms and Diagnosis
Rheumatoid Arthritis Symptoms	Rheumatoid Arthritis Symptoms	Expressing Gratitude and Support
Expressing Gratitude	COVID-19 Vaccination	Prednisone Dosage and Use
Rheumatoid Arthritis Diets	Expressing Gratitude and Support	Rheumatoid Arthritis Information
Prednisone Dosage and Use	RA Diagnosis and Symptoms	Rheumatologist Consultations
Medication Dosages	Healthy Diet and Celiac	RA Diagnosis and Treatment
Doctor Consultations	Prednisone Dosage and Use	Anti-inflammatory Diet
Biologics and Humira	Methotrexate Treatment Effects	COVID-19 Vaccination
Exercise and Physical Therapy	Rheumatologist Appointments	Carpal Tunnel and Finger Issues
Methotrexate Treatment	Methotrexate and Biologics	Medication Dosages

**Table 4:** Top 10 topics extracted from the posts and comments of r/rheumatoidarthritis subreddit across the time-periods.

r/rheumatoidarthritis		
Pre-COVID	COVID	Post-COVID
**Posts**		
Rheumatoid Arthritis and Joint Pain	Rheumatologist and Medication Management	Rheumatoid Arthritis Diagnosis and Symptoms
	Rheumatoid Arthritis Diagnosis	Rheumatoid Arthritis and Inflammation
	Expressing Gratitude and Support	Prednisone and Methotrexate Treatment
	Methotrexate and Side Effects	
**Comments**		
Rheumatologist and Medication Management	Arthritis Diagnosis and Treatment	Rheumatologist and Arthritis Treatment
Rheumatoid Arthritis Diagnosis	Rheumatoid Arthritis Symptoms	Rheumatoid Arthritis and Autoimmune Conditions
Expressing Gratitude and Support	Expressing Gratitude	Expressing Gratitude
Methotrexate and Side Effects	Chronic Illness Therapy	Prednisone Dosage and Tapering
	COVID-19 Vaccination	Biologic Injections
	Rheumatoid Arthritis and Autoimmune Diseases	COVID-19 and Flu Vaccines
	Autoimmune Diets	Hand and Wrist Support
	Prednisone Dosage and Use	Anti-inflammatory Diets
	Biologics	Diagnosis and Medical Consultation
	Methotrexate Treatment	Medication Dosages

Pre-COVID discussions revolved around RA diagnosis, pain management, and medication side effects, frequently mentioning treatments like methotrexate and prednisone. During COVID-19, concerns about RA management increased, with discussions on vaccination, immune responses, and treatment disruptions. Many posts reflected anxieties over drug availability and a shift to telehealth services. Post-COVID, conversations focused on chronic pain and prolonged medication side effects, with new conditions like carpal tunnel syndrome, likely due to increased remote work, becoming topics of discussion.

The topic analysis revealed several recurring themes across the subreddits and time periods examined in this study. The most prominent topic was “Rheumatoid Arthritis (RA) Diagnosis and Symptoms”, indicating that discussions remained relevant and centered on RA-related issues. “Medications for RA” was another frequently discussed topic, with common references to drugs such as methotrexate, prednisone, and biologics, focusing on dosage, side effects, and medication combinations. Conversations about “Diets for Autoimmune Diseases and Anti-Inflammatory Foods” highlighted foods believed to alleviate RA symptoms, along with those considered to worsen them. A recurring and positive theme was “Expressing Gratitude and Support,” where commenters frequently thanked one another and provided a supportive community.

The “COVID-19 Vaccination” topic gained prominence during the pandemic. It persisted afterward, with discussions focusing on the impact of COVID-19 on RA symptoms and the interaction between RA medications and the virus or vaccines. However, post-pandemic, “Carpal Tunnel and Finger Issues” emerged in r/rheumatoid, while “Hand and Wrist Support” appeared frequently in r/rheumatoidarthritis, possibly due to increased computer usage during the work-from-home period. “Rheumatologists and Consultations” were also commonly discussed, with users sharing concerns about the long waiting times and experiences with changing rheumatologists. Finally, Exercise and Physical Therapy” appeared frequently, with users discussing the benefits of physical activities, such as yoga, in managing RA symptoms. These findings suggest that discussions within RA-related subreddits consistently cover the key aspects of living with and managing RA, addressing both medical and lifestyle-related concerns.

### 3.4 Comparative analysis of subreddits

A comparative analysis of the two subreddits, r/rheumatoid and r/rheumatoidarthritis, revealed a high level of similarity between them in terms of content and discussions. The sentiment analysis for the subreddits followed a similar trend, with the majority as positive, followed by negative (Fig 4A). The emotion analysis is also homologous for the subreddits, with joy being the dominating emotion followed by sadness and fear (Fig 4B). The word clouds demonstrate that the most frequently used words are identical in both subreddits, words like “medication”, “doctor”, “help” and “side effects” are prevalent in both subreddits (Fig 4C, 4D).

Additionally, the topic analysis indicates that the top 10 topics are nearly identical for both communities. It highlights the diverse concerns and shared experiences of people with rheumatoid arthritis (RA). As expected, the most common theme across r/rheumatoid and r/rheumatoidarthritis subreddits was **arthritis symptoms and treatments**, with posts frequently revolving around diagnosis, medications, and consultations with rheumatologists. These discussions reflect the community’s focus on understanding and managing their condition. The impact of the COVID-19 pandemic appeared evident as **COVID-19 and flu vaccination** is a prominent topic in both the subreddits, expressing concerns about staying protected while having a compromised immune system, especially with RA. Discussions revolving around **prednisone usage and steroid management** were also common, with members sharing their experiences with reduced dosage and experiences with side effects. The subreddits provided a space for **gratitude and support**, with users thanking one another for advice and celebrating small victories in their RA journeys. Another commonly observed theme was the discussion about the drug **methotrexate**, where members shared concerns about side effects, dosage adjustments, and long-term use. Overall, these discussions illustrate a dynamic and compassionate subreddits where members provide mutual support, share experiences, and acknowledge each other’s experiences in navigating the complexities of RA.

### 3.5 Drug mention analysis

**Fig 5:**
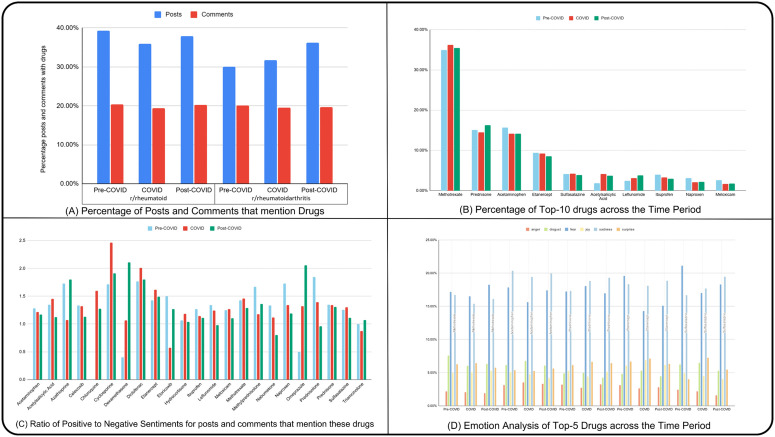
Quantitative and sentiment analysis of rheumatoid arthritis drug discussions. (A) Percentage of posts and comments for the subreddits r/rheumatoid and r/rheumatoidarthritis that discuss about drugs for Rheumatoid Arthritis (B) Percentage of the 10 most discussed drugs across the time-periods (C) Ratio of Positive posts and comments and Negative posts and comments that mention the drug. (D) Emotion analysis of the 5 most discussed drugs across the time-periods.

Our analysis of drug-related mentions in the r/rheumatoidarthritis subreddit across the pre-COVID, COVID, and post-COVID periods revealed significant shifts in the volume and sentiment of discussions. Fig 5A shows the percentage of posts and comments mentioning drugs. Accordingly, a higher proportion of posts were observed compared to comments in all three periods. Notably, drug-related mentions in the r/rheumatoidarthritis subreddit have increased over time. Methotrexate was consistently the most discussed drug across all periods, as shown in Figure B, accounting for approximately 40% of mentions (Fig 5B). Other frequently discussed drugs include prednisone, acetaminophen, and etanercept. Among these drugs, discussions related to methotrexate demonstrated an increase during the COVID period. However, mentions of other drugs like acetaminophen, sulfasalazine, naproxen and meloxicam were less frequent but remained relatively stable across the periods. Discussions on certain drugs like ibuprofen and etanercept decreased over time.

Sentiment analysis revealed variations in the ratio of positive to negative sentiments for posts and comments mentioning different drugs (Fig 5C). Certain drugs, including Methotrexate, Cyclosporine, Diclofenac, Etanercept, Hydrocortisone and Sulfasalazine, exhibited a higher positive-to-negative ratio during the COVID period, suggesting a connection between the drugs and COVID. On the other hand, Acetaminophen and Prednisone showed relatively balanced sentiment ratios across all periods.

Similarly, emotional analysis (Fig 5D) further highlighted differences in the emotional tone of discussions. Across all five drugs examined (Methotrexate, Acetaminophen, Prednisone, Etanercept, and Sulfasalazine), the proportion of mentions remained broadly consistent across the pre-, during-, and post-COVID periods (S2 Fig in S1 File). Fear and sadness were the dominant emotions associated with all drugs, but a clear temporal shift was observed. In the pre-COVID period, fear was more prevalent than sadness for most drugs, whereas during and post-COVID, sadness surpassed fear, indicating an emotional transition in user discussions as the pandemic progressed. Additionally, surprise showed a small increase during the COVID period before returning toward baseline post-COVID.

The analysis of topics extracted from posts and comments discussing drugs highlights notable shifts in community focus across the pre-COVID, COVID, and post-COVID periods. (S3 Table in S1 File). Pre-COVID conversations primarily focused on arthritis diagnosis and pain management, with common themes including using drugs, including prednisone for inflammation, methotrexate (MTX) dosage and treatment, and the impact of cold sensitivity on symptoms. However, during the COVID period, discussions changed to reflect pandemic-related concerns, such as the implications of COVID-19 vaccination for immune-compromised individuals, immune response, and adjustments to medication regimens, including methotrexate administration and prednisone dosage. Interestingly, in the post-COVID discussions, the focus moved to long-term RA management, emphasizing topics like methotrexate effects and administration, prednisone tapering, and alternative symptom relief strategies such as hot-cold therapy. Notably, the most common theme across all periods included methotrexate usage and its side effects, which transitioned from general discussions to specific concerns about administration and long-term impacts. Thus, our analysis highlights the dynamic nature of drug-related discussions within the RA community and highlights how the COVID-19 pandemic influenced patient perspectives on medication usage and management.

### 3.6 Hyperlink-based analysis

**Fig 6:**
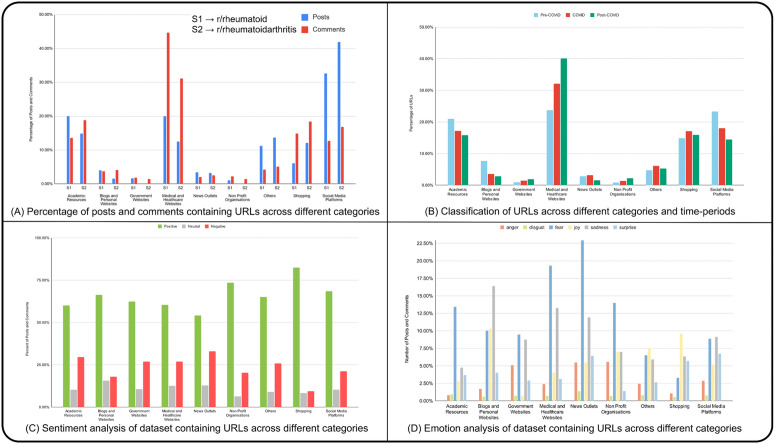
Analysis of URL-containing posts and comments in r/rheumatoid and r/rheumatoidarthritis subreddit. (A) Percentage of posts and comments containing URLs across different categories in r/rheumatoid (S1) and r/rheumatoidarthritis (S2). Categories include Academic Resources, Blogs and Personal Websites, Government Websites, Medical and Healthcare Websites, News Outlets, Non-Profit Organizations, Others, Shopping, and Social Media Platforms. (B) Proportional distribution of URL categories across pre-COVID, COVID, and post-COVID periods in both subreddits, reflecting relative rather than absolute frequencies. (C) Percentage of sentiment distribution (positive, neutral, negative) in URL-containing posts and comments. (D) Percentage of emotion distribution (anger, disgust, fear, joy, neutral, sadness, surprise) in URL-containing posts and comments.

The hyper-link analysis identified 6,717 URLs in posts and comments, consisting of 1,814 unique domains. URL categorization revealed that posts were mainly linked to social media, medical, healthcare, and academic resources (Fig 6A), indicating that users frequently referenced other posts or comments and initiated discussions based on new RA information. Comments primarily included URLs from medical and healthcare websites, followed by shopping and academic sites, suggesting commenters provide reliable information and recommend products to alleviate RA symptoms. Manual validation of the categorised URLs demonstrated substantial agreement with the GPT-4 classifications, with a Cohen’s kappa of 0.90, indicating that the automated categorization was highly accurate and dependable.

The occurrence of URL categories over time showed an increased prevalence of shopping, and news websites during the COVID-19 pandemic (Fig 6B). This trend is likely due to the emergence of COVID-19 and RA information on academic and social media platforms and increased online shopping during lockdowns. Medical, healthcare, government, and non-profit website categories exhibited steady growth, possibly reflecting greater demand for trustworthy information amid heightened awareness of unreliable sources during the pandemic. Academic resources, social media, and blog posts all showed a decreasing trend of hyperlink mentions, with a sharper decline from Pre-COVID to COVID than from COVID to Post-COVID.

Sentiment analysis of the associated URLS indicated that posts and comments were generally more positive. Categories like medical, healthcare, academic resources, and news outlets showed higher negative sentiments (Fig 6C), likely due to discussions on medication side effects or COVID-19’s impact on RA. Conversely, shopping, blogs, social media and personal websites displayed more positive sentiments, often providing support, motivation, or helpful information.

Emotion analysis revealed dominant emotions of fear, sadness, joy, and surprise in posts and comments with hyperlinks. Notably, Joy was prevalent in shopping, blogs, and personal websites, while medical, news, non-profit organisations and academic sites had higher instances of fear and sadness. Social media also showed notable surprised emotions. Thus, our 3-phases temporal analysis shows that compared to non-COVID phases, during the COVID-19 pandemic there is an increased reliance on social media, medical, and academic resources.

## 4. Discussion and conclusion

This study analyzed the evolving nature of rheumatoid arthritis (RA) patient discussions on Reddit across the pre-COVID, COVID, and post-COVID periods (2018–2024) using sentiment analysis, topic modeling, and information-sharing behaviors. Building on the latter, our study captured changes in concerns, emotions, and shared themes during a globally disruptive period, thereby providing insights into the role of online communities in chronic disease management.

Driven by our research questions, our analysis reveals a significant temporal shift in Reddit discourse. Specifically:

In response to R1, emotional expression intensified during the COVID-19 phase, with marked increases in fear, sadness, and uncertainty in posts, contrasted by empathetic, reassurance-driven comments. While post-COVID discussions showed reduced emotional distress and a return to more informational exchanges.

Addressing R2, thematic analysis demonstrated stable core concerns related to diagnosis, symptoms, and treatment across all periods, alongside pandemic-specific expansions into mental health, community support, and holistic management strategies such as exercise and diet.

With respect to R3, medication-related discussions remained dominant, with Methotrexate consistently central, while COVID-19 prompted shifts toward drugs associated with immunosuppression and infection risk; concurrently, hyperlink-sharing peaked during the pandemic and favored credible medical sources, reflecting heightened information-seeking under healthcare disruption. Together, these findings highlight how external crises reshape online patient discourse, influencing emotional expression, treatment priorities, and digital health behaviors.

Previous studies often overlooked the distinction between posts and comments, combining them into a single entity and failing to account for the unique dynamics of initiating discussions versus responding to them. Our study addresses the above-mentioned limitation by analyzing posts and comments separately, offering deeper insights into public health trends and individuals’ perceptions of people living with RA [53]. Moreover, Reddit’s subreddit structure, moderation, and voting mechanisms foster high-quality, focused discussions, making it more effective for social network analysis than other platforms such as Twitter [54]. Our analysis revealed significant trends in user engagement during the COVID-19 pandemic, consistent with earlier findings on Reddit’s COVID activity [55] and the global surge in Internet usage [56]. Furthermore, external events, such as the Reddit API controversy, influenced user behavior, highlighting the impact of platform changes on online communities.

The notable rise in subreddit activity during COVID-19 likely reflects increased reliance on online communities rather than changes in RA incidence. Lockdowns and limited access to rheumatology services drove patients to seek information and support digitally, alongside the expansion of telemedicine. This aligns with evidence of disrupted rheumatology services during the pandemic [57,58], as digital platforms became crucial spaces for support and information exchange during healthcare instability.

Topic modeling highlighted Reddit’s role in providing practical knowledge and emotional support to patients with RA. Dominant themes such as Diagnosis, Symptoms, and Treatment show their capacity for specific information. Expressions of gratitude and support highlight the emotional value of community encouragement. Discussions on Medications reveal complexities in managing treatments such as Methotrexate and Biologics, stressing the need for healthcare professionals to engage with these platforms to correct misinformation and address concerns. Conversations on diet, exercise, and yoga suggest a growing interest in holistic management, aligning with evidence of their benefits in reducing inflammation and improving the quality of life of patients with RA [59]. A study by Reuter et al., examining rheumatoid arthritis (RA) conversations on Twitter during the COVID-19 crisis revealed themes comparable to those found in our investigation, such as COVID-related discussions, the significance of exercise, and the importance of community backing [13]. Both investigations emphasized caution in verifying medical information, which aligns with our findings. However, while Reuter et al. used a manual topic extraction method susceptible to bias and limited scalability, our study utilized the NLP using BERTopic NLP library, ensuring reproducibility and the ability to handle larger datasets, thus offering a more thorough examination of online discourse. These outcomes highlight how moderated online communities can meet patients’ informational and emotional needs, while also providing healthcare professionals with valuable insights to better interact with these platforms, encourage accurate information dissemination, and shape patient-centered care approaches.

Approximately 40% of discussions centered on medications, reflecting the medical nature of these online forums. Methotrexate was the most frequently mentioned drug, consistent with its central role in RA treatment [60]. Discussions also highlighted concerns about the cardiovascular and renal risks associated with NSAIDs like Celecoxib and Naproxen [61,62]. During the pandemic, specific benefits were observed for medications such as Prednisone and Acetaminophen. However, the focus shifted to drugs like Cyclosporine and Etanercept, which were being considered for their potential to reduce COVID-related risks [63,64]. The heightened engagement on Reddit during COVID-19, which mirrors trends in other studies [56], further suggests that external events drive user participation and shift treatment priorities. Medication-related posts often expressed fear and sadness regarding treatment effectiveness and side effects, while joy was associated with successful treatments, underscoring the emotional context of RA management.

Hyperlink analysis revealed that the majority of shared links were related to medical and healthcare websites, reflecting users’ emphasis on credible information. The “No Unsubstantiated Information” rule within the RA subreddits likely contributed to the focus on reliable sources. This trend was especially evident during the COVID-19 pandemic when practical advice on daily living aids, such as gloves and massage machines, gained prominence [65]. Fewer hyperlinks were shared post-COVID, in line with the reduced online shopping behavior observed during the pandemic. Social media links, particularly from Reddit (48.86%) and YouTube (23.16%), are widely shared. YouTube links, which often provided yoga and exercise tutorials or personal RA experiences, also played a significant role in disseminating both practical and emotional support. Further, the number of medical and academic papers discussing RA treatment side effects, often with negative sentiments, has risen.

Our findings suggest significant clinical implications, as they highlight patients’ growing use of online platforms to navigate uncertainty, seek reassurance, and interpret disease information. Understanding these patterns can help healthcare providers anticipate common concerns, misconceptions, and unmet informational needs in digital communities. By incorporating these insights, offering clear explanations, and guiding patients toward trustworthy resources, hospitals and doctors can enhance patient communication and awareness [66,67].

Despite the valuable insights, our study has limitations. First, the findings are based exclusively on Reddit, which may not be fully representative of the broader RA population. Reddit’s primary user base is aged 18–49 [68], while RA mainly affects those aged 30–60 [69,70]. The anonymity of Reddit makes it difficult to determine the precise age distribution of users in our dataset. Nonetheless, some users have self-reported ages between 14 and 80, with an average age of 35. It is important to exercise caution when generalizing these findings to the broader RA population, as the age distribution in our dataset may not accurately reflect this demographic. Additionally, the predominance of U.S.-based and English-speaking users limits the generalizability of our results to non-English-speaking and global RA communities. Lastly, the self-reported nature of Reddit discussions lacks clinical precision, emphasizing the need for complementary data sources to enhance the robustness of our findings.

In conclusion, our longitudinal analysis of RA-related online Reddit underwent significant transformations across the pre-COVID, COVID, and post-COVID periods. These changes were reflected in shifts in emotional states, treatment concerns, and information-seeking behaviors. Online communities served not only as platforms for emotional expression but also as sources of peer support and reliable information, particularly during periods of healthcare disruption. By linking these patterns to patient-centered care, our study underscores the value of integrating digital insights into clinical practice, psychological support, and public health communication. Despite the demographic limitations of Reddit, our findings highlight the importance of using online patient narratives to inform adaptive, empathetic, and evidence-based approaches to chronic disease management.

## Supporting information

S1 File Supporting Information. Contains S1 Table (statistics of the raw Reddit dataset), S2 Table (examples of posts annotated with anger, disgust, fear, joy, neutral, sadness, and surprise emotions), S3 Table (topics extracted from drug-related posts and comments), S1 Fig (comparative word clouds from r/rheumatoid and r/rheumatoidarthritis across pre-COVID, COVID, and post-COVID periods), and S2 Fig (percentage of mentions of commonly discussed drugs across pre-COVID, COVID, and post-COVID periods). (DOCX)
