# Supplementary material for: Temporal evolution of digital health communication in Rheumatoid Arthritis: A longitudinal NLP analysis of reddit discussions (2018–2024)
Source: PLoS One. 2026 Jan 20;21(1):e0341006. doi: 10.1371/journal.pone.0341006 (PMC12818618; doi:10.1371/journal.pone.0341006)
Supplement: S1 File — (DOCX) [file pone.0341006.s001.docx]

**Temporal Evolution of Digital Health Communication in Rheumatoid Arthritis: A Longitudinal NLP Analysis of Reddit Discussions (2018–2024)**

Naisarg Patel^1^, Rajesh Sharma^2,3^, Prakash Lingasamy^4,5^, Vino Sundararajan^1^, Sajitha Lulu S*^1^, Vijayachitra Modhukur^4^

1. Integrative Multiomics Lab, School of Bio Sciences and Technology, Vellore Institute of Technology, Vellore – 632014, Tamil Nadu, India
2. School of AI and Computer Science, Plaksha University, Punjab, India
3. Institute of Computer Science, University of Tartu, 51009 Tartu, Estonia
4. Department of Obstetrics and Gynecology, Institute of Clinical Medicine, University of Tartu, L. Puusepa 8, 50406 Tartu, Estonia
5. Nalam Biosciences OÜ, Tartu, Estonia

***Corresponding author:**

Dr. Sajitha Lulu S, Integrative Multiomics Lab, School of Bio Sciences and Technology, Vellore Institute of Technology, Vellore – 632014, Tamil Nadu, India

**Email:** [ssajithalulu@vit.ac.in](mailto:ssajithalulu@vit.ac.in)

**This file includes:**

**S1 Table:** Statistics of the raw dataset extracted from Reddit

**S2 Table:** Examples of posts annotated as anger, disgust, fear, joy, neutral, sadness, and surprise emotions

**S3 Table:** Topics extracted from the posts and comments that discussed drugs across the time period

**S1 Fig:** Comparative word clouds from subreddits r/rheumatoid and r/rheumatoidarthritis, visualized across three time periods: pre-COVID, during COVID, and post-COVID

**S2 Fig:** Percentage of mentions for commonly discussed drugs (Methotrexate, Acetaminophen, Prednisone, Etanercept, and Sulfasalazine) across Pre-COVID, COVID, and Post-COVID periods.

**Supplementary Table 1:** Statistics of the raw dataset extracted from Reddit

| **Subreddit** | **r/rheumatoid** | | **r/rheumatoidarthritis** | | **Total** | |
| --- | --- | --- | --- | --- | --- | --- |
| **Time** | **Posts** | **Comments** | **Posts** | **Comments** | **Posts** | **Comments** |
| Pre-COVID | 2970 | 35731 | 400 | 2800 | 3370 | 38531 |
| COVID | 9097 | 105206 | 4258 | 41482 | 13355 | 146688 |
| Post-COVID | 6032 | 74174 | 4043 | 43576 | 10075 | 117750 |
| **Total** | **18099** | **215111** | **8701** | **87858** | **329769** | |

**Supplementary Table 2:** Examples of posts annotated as anger, disgust, fear, joy, neutral, sadness, and surprise emotions

| **Emotion** | **Total posts (Percent)** | **Examples** |
| --- | --- | --- |
| **Anger** | 8,873 **(2.99%)** | - You beat me to it. Unless you're actually whincing in pain, people just don't get it. Makes me angry. - Yes!! My doc looked at me like I was crazy but I swear I was full of rage. The smallest things made me want to get physically violent |
| **Disgust** | 17,437 **(5.88%)** | - Kale is totally disgusting and in a smoothie is criminal. - Physically doing them sucks. The smell of alcohol wipes the weird vomit taste in my mouth the day of or next few days. |
| **Fear** | 31,436 **(10.60%)** | - Im starting today and feeling nervous with heart warnings, since these are new, but praying it works!! - It is terrifying to know how bad the side effects can be. Mentally I am also terrified too. Thank you so much for sharing! |
| **Joy** | 32,577 **(10.98%)** | - Im so happy for you enjoy every moment. How wonderful it must feel to be at peace in your own body gives me hope that we can make progress! - I love walking my dog around new places and exploring with him. |
| **Neutral** | 138,361 **(46.63%)** | - Me too I only bring it it up if it’s relevant to the conversation - I would ask for the prednisone. If you have a positive response, they will keep you on a low dose until the plaquenil has had enough time to begin working. |
| **Sadness** | 44,393 **(14.96%)** | - im dealing with some intense dissociation amnesia at the moment (ive also got some wack ass mental illnesses im working thru in therapy atm) and dont entirely remember making this post, but i appreciate your long and well thought out comment! it makes me feel less alone in this confusing process. thank you - I always say I feel bad for Americans, and this is why. That cost me $12 last month in Canada.. Sorry for your loss, RIP Humira.. |
| **Surprise** | 23,616 **(7.96%)** | - I'm surprised by the number of people who've shared they have EDS. I've read about it, and that's got to be a complicated combo. - Shockingly, it did cover most and I have the co-pay card for the balance. (And my insurance is not great by any means) |

**Supplementary Table 3**: Topics extracted from the posts and comments that discussed drugs across the time period

| **Sr. No** | **Pre-COVID** | **COVID** | **Post-COVID** |
| --- | --- | --- | --- |
| 0 | Arthritis Diagnosis and Pain Management | Rheumatologist Visits and Arthritis Treatment | Arthritis Diagnosis and Treatment Management |
| 1 | Prednisone Usage and Inflammation Treatment | NSAIDs and Pain Relief Medications | Methotrexate Usage and Folic Acid Supplementation |
| 2 | Methotrexate (MTX) Dosage and Treatment | Prednisone Dosage and Steroid Use | Enbrel and Injection Therapies |
| 3 | Rheumatoid Arthritis Symptoms and Diagnosis | Methotrexate Side Effects and Administration | Rheumatoid Arthritis Symptoms and Inflammation |
| 4 | Methotrexate Side Effects and Nausea | COVID-19 Vaccination and Immune Response | Prednisone Dosage and Steroid Management |
| 5 | Enbrel Treatment and Injections | Rheumatoid Arthritis Symptoms and Diagnosis | COVID-19 and Immune-Compromised Conditions |
| 6 | NSAIDs and Pain Relief Medications | Methotrexate (MTX) Dosage and Long-term Use | Methotrexate (MTX) Effects and Administration |
| 7 | Cold Sensitivity and Weather Effects | Enbrel Treatment Experiences and Transition | Prednisone Tapering and Arthritis Treatment |
| 8 | Plaquenil and Combination Treatments | Hair Loss Associated with Medications | Pain Relief and Hot-Cold Therapy |
| 9 | - | Pregnancy and Rheumatoid Arthritis Management | Dry Eye Symptoms and Eye Care |


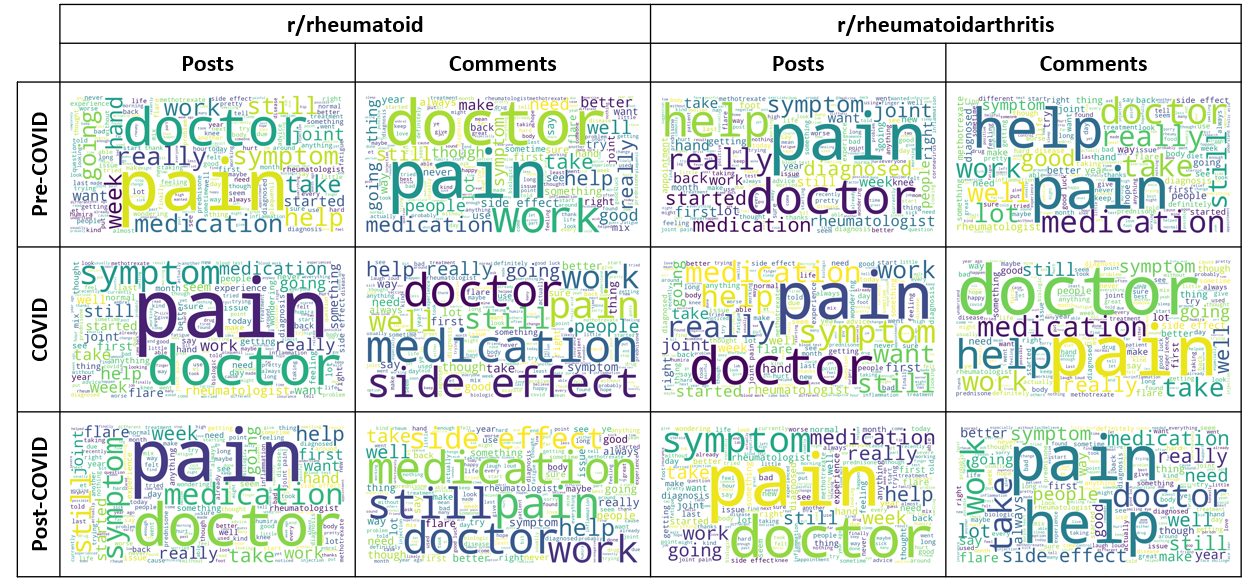


**Supplementary Figure. 1**: Comparative word clouds from subreddits r/rheumatoid and r/rheumatoidarthritis, visualized across three time periods: pre-COVID, during COVID, and post-COVID. The word clouds highlight the most frequently mentioned words within posts and comments for each subreddit during these periods, with font size representing word frequency.


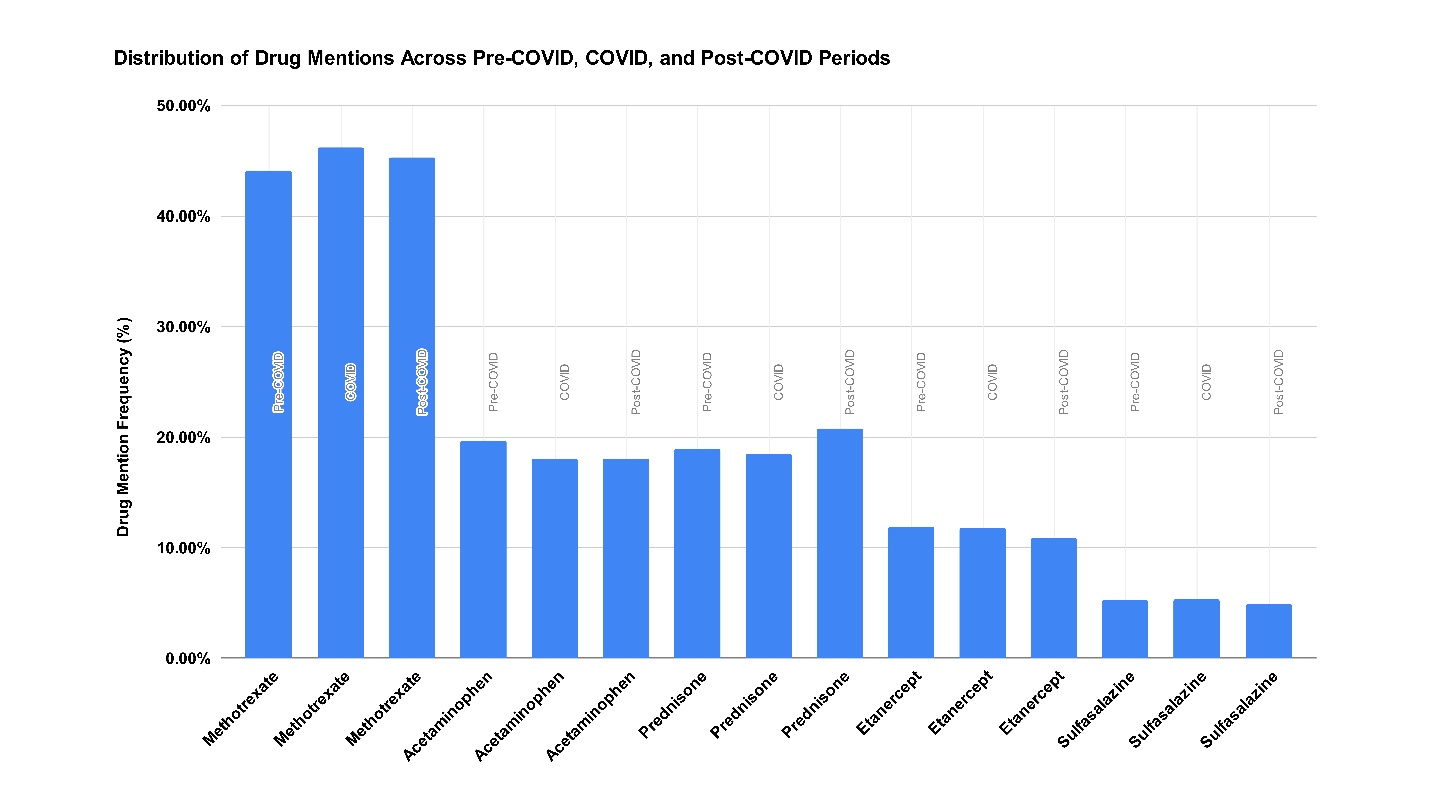


**Supplementary Figure. 2:** Percentage of mentions for commonly discussed drugs (Methotrexate, Acetaminophen, Prednisone, Etanercept, and Sulfasalazine) across Pre-COVID, COVID, and Post-COVID periods.
